# Mind over matter? The cognitive styles of scientific scepticism and paranormal belief

**DOI:** 10.3389/fpsyg.2026.1699045

**Published:** 2026-03-06

**Authors:** Neil Dagnall, Andrew Denovan, Claire Murphy-Morgan, Kenneth Graham Drinkwater, Danny Powell, Nick Neave

**Affiliations:** 1 MMU School of Psychology, Manchester Metropolitan University, Manchester, United Kingdom; 2 LJMU School of Psychology, Liverpool John Moores University, Liverpool, United Kingdom; 3 Northumbria Department of Psychology, Northumbria University, Newcastle upon Tyne, United Kingdom

**Keywords:** analytical-rational, belief in science, cognitive processing preferences, dual processing, intuitive-experiential, paranormal belief

## Abstract

Scientific scepticism, as an epistemic orientation, remains under-researched. This study investigated the interplay between belief in science, supernatural credence, and cognitive processing styles in a sample of 300 participants (*M*_age_ = 45.95, *SD* = 14.32). Traditional (TPB) and New Age (NAP) paranormal beliefs correlated positively with intuitive-experiential measures and negatively with analytical-rational processing indices. Belief in Science showed the inverse pattern of relationships. Latent Profile Analysis (LPA) identified two distinct subgroups: Higher Evidence-based Thinking (HET; 55%), defined by high scientific and low paranormal belief, and Lower Evidence-based Thinking (LET; 45%), characterized by low scientific and high paranormal belief. HET (vs. LET) participants demonstrated significantly greater analytical-rational and lower intuitive-experiential processing. Cognitive rigidity (dogmatism and need for closure) did not differentiate between profiles, suggesting these are belief-neutral characteristics of strongly held convictions. Findings indicated that scientific and paranormal beliefs represent oppositional worldviews associated with distinct, preferred modes of information processing.

## Introduction

The relationship between scientific understanding and paranormal belief remains under-researched (Irwin et al., 2016; Williams et al., 2022). This study addresses this gap by investigating the interplay between belief in science, supernatural credence, and cognitive processing styles. Specifically, the research identifies distinct subgroups based on unique belief configurations and examines how these profiles associate with variations in cognitive style.

### Definition of key theoretical concepts

The central concept in this investigation was scientific scepticism. Consistent with the definitions of Sagan (1979), Normand (2008), and Pigliucci (2009), we define scientific scepticism as an open-minded investigative process that rigorously applies reason to test knowledge and ideas, accepting claims as veridical, only when supported by a compelling body of reliable evidence. Thus, rather than a dogmatic stance that rejects or dismisses data that contradicts prevailing wisdom, this form of scepticism is a rational method based on objective substantiation.

Modern scepticism aligns with the scientific method, which provides a systematic framework for establishing evidence-based conclusions that researchers continuously scrutinize, revise, and refine as new evidence emerges. When applied to paranormal phenomena, sceptics critically assess claims against empirical evidence prior to acceptance (Irwin et al., 2016). Crucially, this methodological scepticism represents an active evaluative process, which is distinguishable from the passive stance of disbelief.

This concurs with Lamont (2007), who demonstrated that disbelievers draw on social and rhetorical mechanisms to maintain rejection of paranormal claims. Lamont et al. (2009) further showed that disbelief involves the active epistemic rejection of a claim, which is fundamentally different from a lack of engagement with or knowledge of a topic. This distinction is central to our investigation of scientific scepticism, as it differentiates between passive unawareness and the active, analytical rejection of paranormal claims. In this context, disbelief is an active cognitive and social process, shaped by group dynamics, cultural narratives, and the perceived credibility of information sources.

Paranormal scepticism assumes that supernatural phenomena have ordinary explanations. This principle underpins anomalistic psychology, which explains how psychological and physical factors create the illusion of paranormal activity where none exists (French, 2001; French and Stone, 2017; Zusne and Jones, 1989). It also reflects a commitment to methodological naturalism, where scholars presume natural causes unless proven otherwise through scientific inquiry. Consequently, psychology rejects paranormal claims because they conflict with established scientific understanding and violate Broad’s (1949) basic limiting principles (Dagnall et al., 2025). These principles rest on fundamental assumptions about reality and causality (e.g., effects cannot precede causes) that align with the scientific method. An alternative to methodological scepticism is the worldview hypothesis (Irwin, 2009; Zusne and Jones, 1982, 1989), which suggests that belief systems function as integrated cognitive structures.

While theorists sometimes partition these concepts into distinct levels of analysis, in the study of individual differences, epistemic stance (process) and worldview (structure) function as a unified lens through which individuals evaluate truth claims. From this perspective, supernatural credence is part of a rationalistic philosophical stance, where reasoning is the principal source and test of knowledge (Irwin, 2017). Within this framework, paranormal validation occurs because it coheres with existing beliefs (Halloran and Kashima, 2004). This approach moves beyond a deficit model of paranormal belief, by viewing these systems as co-existing and competing cognitive structures within individuals.

Worldview is important with respect to belief in the paranormal because it acts as a lens through which individuals interpret reality (Irwin, 2017). This conceptualization shares an intellectual lineage with the philosophical notion of Weltanschauung, which operationalizes worldviews as integrated, self-reinforcing systems of meaning. Hence, believers hold a distinct worldview characterized by high subjectivism and the pursuit of mystical knowledge (Zusne and Jones, 1989). This contrasts sharply with the scientific worldview, which is materialist, rationalist, and reductionist (Trumble, 2013). Consequently, while the paranormal worldview assimilates paranormal beliefs as truths, the scientific worldview relies on evidence and explanations derived from natural laws.

Irwin et al. (2016) operationalized this distinction as rationalistic (tender-minded) versus materialist (tough-minded), contending that paranormal belief stems from subjective interpretation driven by emotion and intuition. Consistent with this, paranormal believers often lack methodological scepticism; tending to seek confirmatory information and discount contradictory evidence (Blanco et al., 2015; Dean et al., 2022; Irwin et al., 2015). This aligns with findings that paranormal belief correlates positively with distrust of science (Clobert and Saroglou, 2015; Corcoran et al., 2023) and negatively with belief in scientific values (Irwin et al., 2015; Pennycook et al., 2020) and science literacy (Fasce and Picó, 2019; Majima, 2015).

### Prior evidence linking belief and cognitive styles

The worldview hypothesis, by operationalizing paranormal belief within general populations as a regular facet of human cognition, represents an important contribution to exploring the differences between sceptics and believers (Irwin et al., 2017). In this context, studies have established that paranormal belief is associated with faulty reasoning (Dagnall et al., 2016a), propensity to cognitive biases (Dean et al., 2022; Williams et al., 2022), preferential thinking style (Drinkwater et al., 2021a, 2021b), and differences in information and perceptual processing (Dagnall et al., 2025; Seymour et al., 2022).

Recognizing that cognitive-perceptual variations within these domains correlate with paranormal belief, researchers have conceptualized supernatural credence as a form of nonpsychotic delusion (Irwin et al., 2012; Drinkwater et al., 2021a, 2021b). This view derives from the observation that believers advocate supernatural phenomena by basing their judgments on emotional appeal, which in turn leads them to overlook contradictory evidence and bypass rational analysis and scepticism (Coltheart et al., 2011).

### Dual-process theory as a conceptual bridge

Noting that believers (vs. non-believers) draw on affect and apply truncated logic to substantiate their faith, investigators have increasingly examined differences from a dual processing perspective (e.g., Aarnio and Lindeman, 2005; Bensley, 2023; Pennycook et al., 2012). This conceptualization proposes the presence of two concurrently operating, but distinct processing systems. While dual models vary in terminology, emphasis, and complexity, this approach provides a robust framework for distinguishing between cognitive and emotion-driven systems in believers and sceptics (Denovan et al., 2024). The cognitive system operates analytically, processing information based on rational principles in a deliberate, demanding, and effortful manner. In contrast, the emotion-driven system is automatic, rapid, and less demanding, relying on intuition, associations, and general heuristics (Williams et al., 2022).

### Cognitive-experiential self-theory

A notable dual-processing framework is Cognitive-Experiential Self-Theory (CEST; Epstein, 1990), which combines principles from psychodynamic, cognitive, and learning theories (Epstein, 1994). CEST proposes the existence of analytical-rational and intuitive-experiential systems. The analytical-rational system is intentional, effortful, and because it depends on higher-order cognitive functions, limited in capacity. In contrast, the intuitive-experiential system is fast, automatic, and holistic, basing judgments on affect, emotion, and prior experiences (Epstein et al., 1996). Though these systems operate in parallel, individuals exhibit a dispositional preference for one.

This theoretical conceptualization is operationalized by the Rational-Experiential Inventory (REI), a widely used self-report measure of processing style (Pacini and Epstein, 1999). The REI contains subscales that assess a person’s need for cognition (analytical-rational system) and faith in intuition (intuitive-experiential system). Due to theoretical coherence and established psychometric properties, researchers engaged in dual-processing research prevalently use the REI (Evans, 2008; Martingano and Konrath, 2022). Nonetheless, it is important to note that dual processing is a general theoretical framework, which embraces myriad models such as System 1 (fast, automatic, and intuitive) and System 2 (slow, deliberate, and logical) thinking (Kahneman and Frederick, 2002; Kahneman, 2011).

### Present study

The primary objective of the present study was to explore the interplay between scientific and supernatural worldviews by identifying distinct participant profiles based on their unique constellations of belief. By moving beyond the traditional believer versus non-believer dichotomy, we determined how these integrated cognitive-belief structures relate to specific processing styles. To capture the breadth of intuitive-experiential and analytical-rational system features the present study employed a range of self-report instruments. Experiential-intuitive measures collectively assessed the extent to which individuals rely on instinct, affect, and personal experience when processing information. Specifically, deficits in reality testing designate greater reliance on subjective intrapsychic activity over external verification (Lenzenweger et al., 2012). Faith in intuition gaged dependence on instinctual impressions and feelings (Pacini and Epstein, 1999), while the Empathy Quotient assessed rapid, affective, and non-analytical social–emotional processing (Baron-Cohen and Wheelwright, 2004).

Analytical-rational measures evaluated preference or capacity for systematic, logical, and evidence-based thought. Need for cognition appraises reliance on, enjoyment of, and engagement in analytical and logical reasoning (Pacini and Epstein, 1999), and the systemizing quotient gages preference for logical, rule-based understanding (Baron-Cohen et al., 2003). Together, these measures tap into deliberate, effortful cognitive processes central to sceptical evaluation and the desire to engage in scientific inquiry.

Additionally, the study included dogmatism and need for closure as measures of epistemic closing and cognitive rigidity. These constructs reflect the cognitive tendency to depart from/or truncate critical, evidence-based evaluation. Dogmatism denotes cognitive rigidity and lack of openness to diverse viewpoints or information contradicting existing beliefs (Shearman and Levine, 2006). Need for closure denotes motivation for rapid, definitive conclusions (Roets and Van Hiel, 2011). Combined these constructs index resistance to open, critical processing and preference for certainty.

To examine epistemic orientation, individual approaches to validating knowledge (Trumble, 2013; Irwin, 2017), the researchers assessed paranormal belief and belief in science. To further differentiate between types of supernatural credence, the study assessed traditional (e.g., traditional religious belief, and witchcraft) and new age beliefs (e.g., psi, spiritualism and precognition) (Lange et al., 2000). These dimensions added a useful nuance to the measurement of belief since they fulfill distinct adaptive functions. Traditional paranormal belief affords a sense of social control over external events and new age philosophy instills perception of personal control over external events at an individual level (Lange et al., 2000; Drinkwater et al., 2024a).

Belief in science is the extent to which individuals endorse the legitimacy and value of the scientific approach (Farias et al., 2013). Explicitly, individuals are convinced that scientific methods and findings provide accurate and valid insights into reality, and consider science as able to solve problems, explain the world, and answer important questions (Dagnall et al., 2019; Denovan et al., 2024).

Integrating paranormal belief dimensions with belief in science allowed the authors to examine the interplay between scientific and supernatural worldviews in greater depth. This approach facilitated the identification of participant profiles based on unique constellations of belief, moving beyond the traditional believer versus non-believer dichotomy. Such differentiation was necessary because determining the legitimacy of paranormal phenomena is complex. Illustratively, scholars publish positive studies on psi (e.g., telepathy, precognition, and psychokinesis) in respected academic journals (i.e., Society for Psychical Research and American Psychological Association). This scholarly work suggests that the phenomena are scientifically credible.

Finally, this study advanced understanding of scepticism (versus belief) by using a person-centered approach. Although, variable-centered research, which assumes belief varies only by intensity along a single dimension, has robustly linked paranormal beliefs with intuitive-experiential measures, it has limitations. Such work often relies on single-construct measures and assumes outcomes estimate population-averaged associations (Orri et al., 2017). Acknowledging this, the authors explored the coexistence of scientific and paranormal beliefs within individuals using latent profile analysis (LPA). LPA is a statistical technique that identifies unobserved subgroups (profiles) from unique patterns of scores on multiple variables. A key advantage of LPA is the ability to capture belief heterogeneity by considering compound factors (Drinkwater et al., 2021a, 2024b).

### Research hypotheses

Though this study was exploratory, we hypothesized that paranormal believers who scored higher on belief in science (vs. lower in science belief) would score lower on intuitive-experiential and higher on analytical-rational measures. Conversely, profiles exhibiting higher belief in science, but lower paranormal belief would demonstrate higher levels of dogmatism and engage less in analytical-rational processing. These hypotheses addressed methodological scepticism by positing that endorsement of science does not guarantee analytical thinking, suggesting that reliance on intuitive processing undermines a truly sceptical approach, even among those with high scientific belief.

## Method

### Participants

In total, 300 participants completed study measures, mean age (*M*_age_) = 45.95 years (*SD* = 14.32, range of 18–78); 100 males (33%, *M*_age_ = 47.05, *SD* = 13.29, range of 18–78) and 187 females (62%, *M*_age_ = 45.66, *SD* = 14.89, range of 18–77); 8 non-binary (3%, *M*_age_ = 41.25, *SD* = 14.80, range of 19–69); and 5 preferred not to say (2%, *M*_age_ = 42.40, *SD* = 13.28, range of 24–58). Supplementary Table S1 contains additional sample characteristics. The researchers recruited participants via social media (i.e., Facebook; Twitter; now X). To take part, participants had to be at least 18 years of age and provide informed consent by ticking a box on the online survey.

## Materials

This study used a battery of established self-report measures.

### Belief measures

#### Paranormal

The Revised Paranormal Belief Scale (RPBS) (Tobacyk, 2004) comprises 26 items that assess belief in supernatural phenomena. The scale comprises 7 subscales: Traditional Religious Belief (e.g., “I believe in God”); Psi (e.g., “A person’s thoughts can influence the movement of physical objects”); Witchcraft (e.g., “Witches do exist”); Superstition (e.g., “If you break a mirror you will have bad luck”); Spiritualism (e.g., “It is possible to communicate with the dead”); Extraordinary Life Forms (e.g., “The Loch Ness Monster of Scotland exists”) and Precognition (e.g., “Some psychics can accurately predict the future”). Items appear as statements and participants respond using a 7-point scale (1 = strongly agree to 7 = strongly disagree).

Due to issues with factor structure and differential item functioning, Rasch purification produced a two-factor model comprising Traditional Paranormal Beliefs (TPB) and New Age Philosophy (NAP) (Lange et al., 2000). TPB measures customary, paranormal concepts (i.e., devil, heaven, hell, and witchcraft), whereas NAP appraises belief in paranormal abilities (e.g., ability to psychically influence the material world, psychokinesis; and forecast forthcoming events, precognition) and states (e.g., alternate consciousness, astral projection, and spirits) (Drinkwater et al., 2024a). Conceptually these factors correspond to the distinction between ancient/traditional and contemporary/eclectic beliefs, practices, and ways of life.

Psychometrically, the RPBS total (Drinkwater et al., 2017) and two-factor solution have demonstrated good internal consistency (Dagnall et al., 2016a,b). To produce TPB (11-items) and NAP (5-items) dimensions, researchers convert responses from 1–7 to 0–6 and remove items that have previously displayed differential item functioning. Overall scores range between 0 and 156.

#### Science

The Belief in Science Scale BISS (Farias et al., 2013) is a 10-item measure that assesses faith in the value of science as an institution and source of superior knowledge. BISS items appear as statements (e.g., “Science is the most efficient means of attaining truth”) and participants specify the extent to which they agree using a 6-point scale (1 = Strongly Disagree to 6 = Strongly Agree). Total scores range from 10 to 60, with higher scores denoting greater belief in science. The BISS has established validity and internal consistency (Dagnall et al., 2019; Denovan et al., 2024).

### Cognitive-perceptual processing

#### Reality testing

The 20-item subscale of the Inventory of Personality Organization (IPO-RT; Lenzenweger et al., 2012) assessed reality testing. This is the extent to which individuals can discriminate between intrapsychic and external stimuli (see Kernberg, 1996). This conceptualization emphasizes cognitive style over overt psychotic symptoms and features prominently within information-processing accounts of belief generation (Langdon and Coltheart, 2000; Irwin, 2004).

The IPO-RT presents items as statements (e.g., “I have seen things which do not exist in reality”) and participants record their level of endorsement on a five-point Likert-type scale, ranging from 1 (never true) to 5 (always true). Overall scores range from 20 to 100, with higher scores signifying greater tendency to engage with intra-psychic activity (subjective data) and detach from external stimuli (objective data). The IPO-RT has established psychometric integrity (i.e., good internal consistency, test–retest reliability, and construct validity) (Lenzenweger et al., 2012). Researchers frequently use the IPO-RT as a measure of intuitive thinking (e.g., Williams et al., 2022).

#### Rational-experiential processing

The Rational-Experiential Inventory-40 (REI-40; Pacini and Epstein, 1999) is a 40-item instrument that evaluates propensity to engage in rational (20 items) and experiential (20 items) thinking styles. The rationality scale assesses the ability to rely on, enjoy, and engage in analytical and logical reasoning. The experientiality scale measures the capacity to rely on, enjoy, and engage with intuitive impressions and feelings during decision making. Researchers use the rational and experiential subscales to assess the degree to which individuals engage in objective and subjective thinking. The REI presents items as statements (e.g., “I trust my initial feelings about people”) and participants respond to items on a 5-point Likert-type scale (1 = definitely not true of myself, 5 = definitely true of myself). The REI-40 demonstrates good reliability and validity across different populations (Björklund and Bäckström, 2008; Purić and Jokić, 2023).

#### Systemizing and empathy

The Systemizing Quotient Short (SQ-S) and the Empathy Quotient Short (EQ-S) measured differences in cognitive styles. Both derive from the full-scale versions, the Systemizing Quotient (Baron-Cohen et al., 2003) and the Empathy Quotient (Baron-Cohen and Wheelwright, 2004). The SQ-S (25 items) assesses an individual’s drive to analyse and construct systems, specifically the tendency to identify underlying rules, patterns, and cause-and-effect relationships within abstract and concrete systems.

The EQ-S (22-items) measures capacity for empathy. It evaluates cognitive empathy (i.e., ability to understand another person’s thoughts and feelings) and affective empathy (i.e., capacity to respond emotionally to another person’s mental state). Items within the scales appear as statements (e.g., SQ-S: “I am fascinated by how machines work”; EQ-S: “I really enjoy caring for other people”). Participants respond on a 3-point Likert-type scale (“very much agree,” “somewhat agree,” or “disagree”), scored 0 to 2. Total SQ-S scores range from 0 to 50, with higher scores indicating a stronger drive to systemize, and EQ-S scores range from 0 to 44, with higher scores reflecting a greater capacity for empathy. Both the SQ-S and EQ-S possess established psychometric properties (see Baron-Cohen et al., 2003, SQ-A; and Baron-Cohen and Wheelwright, 2004, EQ-S).

#### Dogmatism

The Dogmatism Scale (DOG) (Shearman and Levine, 2006) is an 11-item measure of closed-mindedness (i.e., reluctance to consider information that contradicts existing beliefs). Each item appears as a declaration (e.g., “There is a single correct way to do most things”) and respondents record their answers on a 5-point Likert-type scale ranging from 1 = “Completely Disagree” to 5 = “Completely Agree.” DOG scores range from 11 to 55, with higher scores indicating greater dogmatism. The instrument demonstrates strong psychometric properties (e.g., high internal consistency and validity) (Shearman and Levine, 2006; Von Mohr et al., 2025).

#### Need for closure scale

The Revised Need for Closure Scale (RNCS) (Roets and Van Hiel, 2011) assessed motivation to reach rapid and definite conclusions and is a shortened version of the original Need for Closure scale (NFC; Webster and Kruglanski, 1994). Explicitly, the RNCS comprises 15 items, presented as statements (e.g., “I enjoy having a clear and structured mode of life”), which participants rated on a 6-point Likert-type scale ranging from 1 (completely disagree) to 6 (completely agree). Total scores range from 15 to 90 with higher RNCS totals indicating a greater motivation for cognitive closure. The RNCS is a reliable and valid instrument (Crowson, 2013; Roets and Van Hiel, 2011).

Within the present study measures demonstrated acceptable to excellent internal reliability: RPBS, α = 0.95; NAP, α = 0.94; TPB, α = 0.84; BISS, α = 0.90; IPO-RT, α = 0.87; Rational, α = 0.90; Experiential, α = 0.90; SQ-S, α = 0.85; EQ-S, α = 0.90; DOG, α = 0.75; and RNCS, α = 0.85;

#### Procedure

The researchers collected data using an online survey, which took participants approximately 20 min to complete. First, participants reviewed an online information sheet detailing the study’s purpose, procedures, and ethical considerations. After providing informed consent, they proceeded to the survey, which included measures of scientific and paranormal belief and cognitive processing style. Participants indicated their age and gender and had the option to create a unique codeword for potential data withdrawal. Data collection was anonymous; the researchers recorded no personal identifiers or IP addresses. To address common method variance, the researchers included a confidentiality statement to encourage honest responses, informed participants that answers should reflect individual inclinations and emphasized construct uniqueness via section instructions (see Krishnaveni and Deepa, 2013; Spector, 2019).

The Research Ethics Committee at Northumbria University approved the study (number 36804) and researchers stored electronic data on a password-protected university drive in accordance with University and GDPR guidelines.

#### Analysis

Preliminary analysis examined descriptive relationships. Latent profile analysis (LPA) based on Paranormal Belief subscales (Traditional Paranormal Belief and New Age Philosophy) and Belief in Science scores then established group membership. Analysis utilized Mplus version 8 (Muthén and Muthén, 2018).

LPA computed models with an increasing number of latent profiles (starting with a 1-profile solution) until incorporating additional profiles failed to improve fit. Specific indices included: Akaike Information Criterion (AIC; Akaike, 1987), Bayesian Information Criterion (BIC; Schwarz, 1978), sample-size adjusted BIC (ssaBIC; Sclove, 1987), Lo–Mendell–Rubin-adjusted likelihood ratio test (LMR-A-LRT; Lo et al., 2001), and entropy (Ramaswamy et al., 1993). Higher AIC, BIC, and ssaBIC indicated weaker fit. LMR-A-LRT included a significance test (with 0.05 as the threshold), and entropy scores > 0.8 revealed clear profile separation relative to data (Ramaswamy et al., 1993). Scrutiny of profile differences in relation to processing style measures occurred as a final analytic stage.

## Results

### Descriptive statistics

All univariate skewness and kurtosis values fell between −2 and +2 (Tabachnick and Fidell, 2001). Multiple correlations existed, thus requiring a control for the familywise error rate. Implementation of a sequential approach occurred, including *p*-value ranking with critical *p*-values (Benjamini and Hochberg, 1995). This utilized the 0.05 significance threshold.

Using the Benjamini and Hochberg (1995) procedure, small to large correlations existed between Paranormal Belief (total score), New Age Philosophy, Traditional Paranormal Belief, Belief in Science, Reality Testing, Empathizing, Rational, and Experiential. Need for Closure and Dogmatism demonstrated non-significant associations with the variables used for LPA (aside from Need for Closure with Traditional Paranormal Belief, and Dogmatism with Belief in Science) (Table 1). Gignac and Szodorai’s (2016) guidelines determined correlation strength. Specifically, 0.10, 0.20, and 0.30 represent small, typical, and large associations.

**Table 1:** Descriptive statistics and intercorrelations among all study variables.

Variable	Mean	*SD*	1	2	3	4	5	6	7	8	9	10	11
1. Paranormal belief (total score)	79.84	32.22		0.89**	0.89**	−0.60**	0.08	−0.03	0.55**	−0.14*	0.23**	−0.22**	0.45**
2. New age philosophy	22.64	15.66			0.79**	−0.55**	0.03	−0.03	0.52**	−0.16*	0.20**	−0.22**	0.40**
3. Traditional paranormal belief	10.69	7.82				−0.55**	0.13*	0.01	0.47**	−0.19**	0.19**	−0.28**	0.42**
4. Belief in science	38.49	10.75					0.09	0.20**	−0.34**	0.28**	−0.22**	0.20**	−0.42**
5. Need for closure	53.25	11.54						0.22**	0.22**	−0.08	−0.19**	−0.16*	−0.05
6. Dogmatism	21.47	4.72							0.14*	−0.07	−0.30**	−0.16*	−0.16*
7. Reality testing	48.37	12.40								−0.16*	−0.11*	−0.23**	0.22**
8. Systematizing	54.02	8.48									0.02	0.45**	−0.08
9. Empathizing	51.24	8.30										0.13*	0.42**
10. Rational	75.76	11.35											0.01
11. Experiential	67.12	12.38											

Means displayed are raw total means; **indicates *p* < 0.001.

### Latent profile analysis

Preliminary appraisal of 1 vs. 2-profile solutions indicated superior fit for the 2-profile model, evident from lower AIC, BIC and ssaBIC (Table 2). Subsequently, comparison of 2 vs. 3-profile models suggested better fit for the 2-profile solution, evident from superior entropy and a non-significant improvement, LMR-A-LRT *p* = 0.178.

**Table 2:** Fit of competing latent profile models.

Model	AIC	BIC	ssaBIC	LMR-A	LMR-A *p* value	Entropy
1-profile	3086.51	3108.74	3089.71			
2-profile	2652.25	2689.29	2657.58	423.68	<0.001	0.89
3-profile	2574.62	2626.47	2582.07	82.04	0.178	0.82

AIC, Akaike Information Criterion; BIC, Bayesian Information Criterion; ssaBIC, sample-size adjusted BIC; LMR-A, Lo–Mendell–Rubin-adjusted likelihood ratio test.

**Figure 1:**
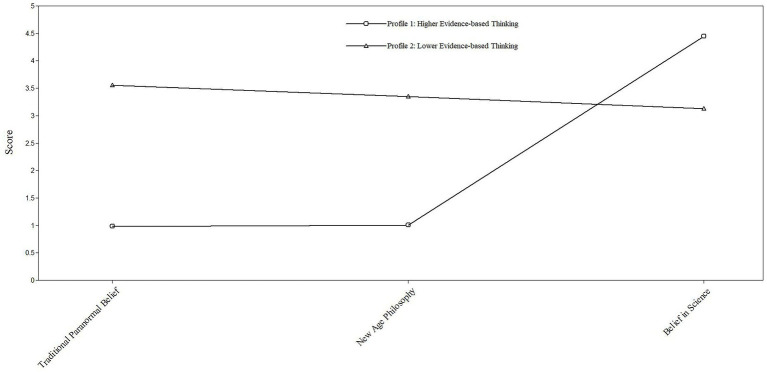
The pattern of mean scores on traditional paranormal belief, new age philosophy, and belief in science as a function of latent profile.

**Figure 2:**
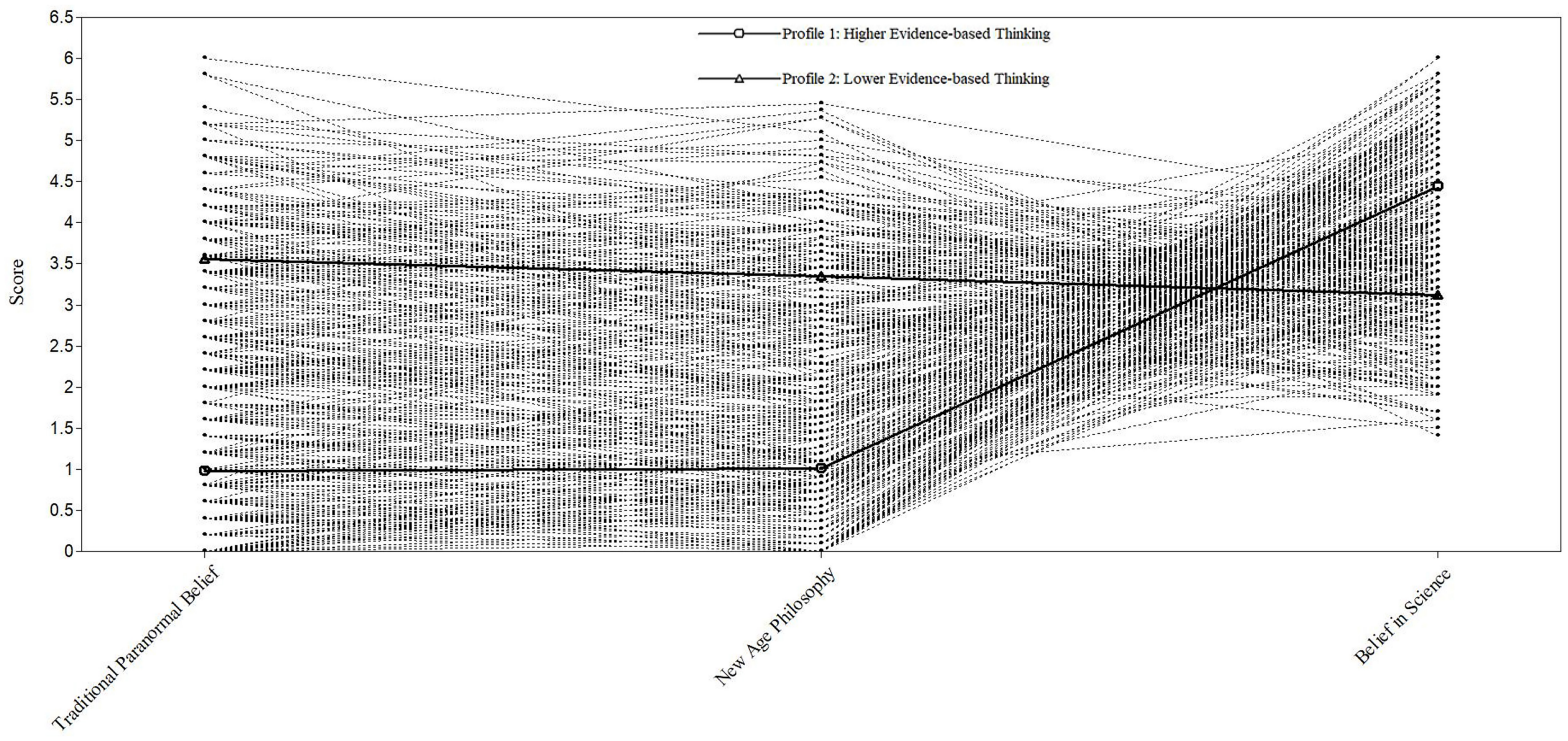
Subject data for each participant within the sample in relation to the latent profiles. Note: Broken lines represent single participant data points; bold lines represent latent profiles.

The 2-profile model (Figure 1) depicts profiles/classes and standardized conditional response means appear in Table 3. Profile 1 (labeled as “Higher Evidence-based Thinking”) comprised 55% (*n* = 165) of the sample and reflected higher scores on Belief in Science, and lower New Age Philosophy and Traditional Paranormal Belief scores. Profile 2 (identified as “Lower Evidence-based Thinking”) included 45% (*n* = 135) and evidenced higher New Age Philosophy and Traditional Paranormal Belief and lower Belief in Science. Comparison with previously reported means occurred to reinforce claims of higher and lower for profiles (cf. Drinkwater et al., 2024a; Hajek and König, 2024). Average latent class probabilities suggested good discrimination (Profile 1 = 0.97, Profile 2 = 0.96). Figure 2 includes subject data for each participant within the sample, demonstrating how the latent profiles emerged from the range of participant responses.

**Table 3:** Standardized conditional response means among profiles.

Measure	Profile 1: Higher evidence-based thinking	Profile 2: Lower evidence-based thinking
Traditional paranormal belief	1.09	3.98
New age philosophy	1.23	4.11
Belief in science	5.22	3.67

### Assessment of latent profiles in relation to processing styles

Multivariate analysis of variance (MANOVA) examined relationships between latent profiles and processing style measures. A significant main effect of group existed, Pillai’s trace = 0.37, *F* (7, 292) = 24.66, *p* < 0.001, η^2^ = 0.37 (large effect size). Significant effects of group occurred concerning Reality Testing, Systematizing, Empathizing, Rational, and Experiential (Table 4). Specifically, Profile 2 scored significantly higher than Profile 1 on Reality Testing (*M* = 54.63 vs. 43.26), Empathizing (*M* = 53.54 vs. 49.34), and Experiential (*M* = 73.0 vs. 62.30), and significantly lower on Systematizing (*M* = 52.53 vs. 55.23) and Rational (*M* = 73.50 vs. 77.60).

**Table 4:** Analysis of Variance (ANOVA) between Profile 1 and Profile 2.

Variable	*df*	*F*	*η* ^2^	*p*
Need for closure	1, 298	0.46	0.01	0.496
Dogmatism	1, 298	3.29	0.01	0.071
Reality testing	1, 298	79.15	0.21	<0.001
Systematizing	1, 298	7.70	0.03	0.006
Empathizing	1, 298	20.25	0.06	<0.001
Rational	1, 298	10.01	0.03	0.002
Experiential	1, 298	67.78	0.19	<0.001

Profile 1 = Higher Evidence-based Thinking; Profile 2 = Lower Evidence-based Thinking.

## Discussion

This study investigated relationships between belief in science, supernatural credence (Traditional Paranormal Belief and New Age Philosophy), and differences in cognitive processing style. The central aims of the study were to produce cognitive profiles based on patterns of concurrent scientific and paranormal belief (i.e., identify distinct subgroups within the population) and reveal associations between subgroup membership and cognitive style.

Consistent with our definition of scientific scepticism as an active epistemic orientation, Traditional Paranormal Beliefs (TPB) and New Age beliefs (NAP) correlated highly (*r* = 0.79) and were similarly negatively associated with Belief in Science (TPB, *r* = −0.55; NAP, *r* = −0.55). This pattern of correlations designated that paranormal belief types were complementary in nature and divergent from scientific orientation. Critically, the Latent Profile Analysis (LPA) identified two distinct groups that represent competing worldviews rather than a simple lack of knowledge.

Furthermore, TPB and NAP were similarly positively associated with intuitive-experiential measures (i.e., proneness to Reality Testing Deficits, Empathizing, and Experiential Processing) and negatively associated with analytical-rational measures (i.e., Systematizing and Rational Processing). Conversely, Belief in Science showed positive relationships with analytical-rational measures and negative relationships with intuitive-experiential measures. These outcomes supported Irwin’s et al. (2016) assertion that paranormal and scientific beliefs are oppositional (see also Irwin et al., 2017).

Results aligned with prior research, which reports that paranormal and scientific beliefs are affiliated with distinct cognitive processing styles. Paranormal belief involves a greater reliance on experiential and emotional processing (Drinkwater et al., 2022), where individuals prioritize internal, subjective information and affective responses over objective, external data (e.g., Aarnio and Lindeman, 2005; Dean et al., 2022; Pennycook et al., 2012). In contrast, belief in science places a stronger emphasis on objective verification and less emphasis on subjective and emotional factors (e.g., Corcoran et al., 2023; Dagnall et al., 2019). In this context, findings supported the notion that paranormal beliefs reflect a rationalistic (tender-minded) worldview allied to subjective interpretation and intuition, whereas belief in science aligns with methodological scepticism and a materialist (tough-minded) worldview, appraising claims based on objective substantiation and evidence-based conclusions.

Regarding epistemic closure and cognitive rigidity, Need for Closure and Dogmatism were positively correlated (*r* = 0.20), indicating that a preference for certainty aligns with reluctance to consider information that contradicts existing beliefs. These traits were differentially associated with belief types. A small positive correlation between Traditional Paranormal Beliefs (TPB) and Need for Closure (*r* = 0.13) suggests that individuals endorsing stronger TPB are more likely to seek definitive answers and avoid ambiguity.

In contrast, the medium positive association between Belief in Science and Dogmatism (*r* = 0.22) implies that commitment to scientific understanding may involve a degree of intellectual inflexibility. This finding provides a significant cognitive nuance. Particularly, it suggests that rather than being exclusive to supernatural credence, dogmatism is a belief-neutral trait that characterizes the intensity with which any worldview is held. This hints at a potential distinction between embracing open inquiry and rigid adherence to established scientific conclusions. Overall, the absence of consistent relationships between beliefs and these traits suggests that epistemic closure and cognitive rigidity are typical characteristics of strongly held beliefs, regardless of content, and warrant further consideration in future research.

Based on paranormal (Traditional and New Age) and science beliefs Latent Profile Analysis (LPA) identified two distinct profiles, Higher Evidence-based Thinking (HET, 55% of sample) and Lower Evidence-based Thinking (LET, 45% of sample). Identification of two distinct cognitive-perceptual processing profiles provided an appropriate framework for understanding the interaction between paranormal and scientific beliefs. This solution captured the primary division between objective and subjective preferences in knowledge acquisition, on the basis of profile scores being higher or lower than previously reported averages. This parsimonious model distinguishes between major forms of knowing and in doing so aligns with dual-processing distinctions.

Ascertaining how the sample split corresponds to reported levels of belief is challenging because academic surveys and polling companies ask different questions and use varying indices of paranormality. Noting these issues, a recent US Gallup poll is relevant: cluster analysis identified two groups, 34% who are open to paranormal concepts and endorse multiple phenomena, and 66% who are sceptical, believing in only one on average (Yi and Hogenboom-Jones, 2025). Given these measurement issues, a more informative indicator of paranormality is subjective experience since personal encounters often precede or reinforce beliefs and provide direct insight into the lived reality of believers. Such experiences involve the ascription of supernatural causation to observed anomalies (Lange et al., 2019). Studies using UK-based samples report similar percentages of paranormal experience to the LET (vs. HET) profile in the present study (Haraldsson and Houtkooper, 1991; Dagnall et al., 2016b).

### Dispositional cognitive profiles

Higher scores on Belief in Science and lower scores on Paranormal Belief characterized the HET profile, whereas the LET profile showed higher Paranormal Beliefs and lower Belief in Science. Analyses found that profile membership was associated with differences in cognitive style. HET (vs. LET) scored higher on Systematizing and rational processing (i.e., Need for Cognition) and lower on Reality Testing Deficits, Empathizing, and Experiential (i.e., Faith in Intuition) processing. Thus, HET displayed an analytical-rational style and LET an intuitive-experiential style. These findings supported the hypothesis that distinct patterns of paranormal and scientific beliefs correspond with variations in cognitive processing, consistent with dual-processing models of belief.

Explicitly, the HET profile embodied a methodological worldview. For these individuals, the active rejection of the paranormal was not merely an absence of belief, but a commitment to an evidence-based epistemic stance. While the HET group showed a degree of dogmatism, suggesting that any strongly held worldview can become rigid, their overall reliance on systemizing and rational processing indicated a preference for analytical verification over subjective intuition. This reinforces our conceptualization of scientific scepticism as an active, analytical process rather than a passive lack of engagement with the topic.

The LPA solution designates that paranormal credence and scientific scepticism, whilst independent constructs, represent common ways of engaging with the world. These findings align powerfully with dual-process theories of cognition, confirming that differences in the habitual use of System 1 (intuitive) versus System 2 (analytical) processing are foundational to how individuals evaluate extraordinary claims. The division of participants into HET and LET profiles indicates that these styles reflect preferred, predominant modes of information processing, cognitive lenses, through which individuals interpret reality.

The division of participants into the Higher and Lower Evidence-based Thinking profiles, based on dispositional measures (i.e., beliefs), indicates that styles reflect preferred, predominant, modes of information processing, which operate as cognitive lens through which individual interpret and validate incoming information.

We conclude that profiles are stable because they derive from established self-perpetuating belief systems that are resistant to change. Nonetheless, future work could test this assumption by examining where contextual factors (e.g., cognitive load, emotional priming, or conditions of uncertainty) impact upon the expression and efficiency of processing styles.

Profile membership was not differentially associated with Dogmatism or Need for Closure. This indicates that cognitive rigidity is a general property of strongly held beliefs, regardless of content, making fervent scientific and paranormal adherents equally likely to dismiss challenges to their established views and reduce ambiguity through rapid, decisive decision-making. Future research, however, should investigate how Dogmatism and Need for Closure interact with the distinct System 1/System 2 dispositions to contribute to the formation and maintenance of specific worldviews.

### Dual-process theory and belief formation: implications

The identification of two profiles, Higher and Lower Evidence-based Thinking, was an important contribution to the dual-process literature. Previous research reporting differences between intuitive (System 1) and analytical (System 2) processing has extensively employed the variable-centered approach (e.g., correlation or regression), which focuses on single belief measures. This methodology fails to capture the complexity of concurrently existing scientific and paranormal beliefs. Using LPA to examine the constructs in combination addressed this limitation. Although analysis failed to extend population subgroups beyond a two-profile solution outlined within the established dual process dichotomy, segregation was an empirical, data-driven outcome. This suggests that epistemological worldview affiliates with preferential thinking style. Particularly that internally coherent cognitions influence acceptance and rejection of paranormal (i.e., sceptical inclination). This advances the investigations in the field variable-centered analysis of cognitive measures to the consideration of belief-generating cognitive frameworks.

### Limitations

Although this study afforded novel theoretical insights into the interplay between scientific and paranormal beliefs and their relationships with cognitive processing styles, the authors acknowledge study limitations.

Firstly, the researcher collected data using self-report measures. This method, though used widely in belief-based research is vulnerable to response bias. This can occur in the form of common method variance, where the use of the same method artificially inflates or deflates observed relationships. Since the research team has experience of self-report measures the study employed methodological devices to mitigate its impact. Specifically, instructions to participants emphasized the distinct nature of each survey section and construct differences, and the survey employed a range of response scales. These procedures have previously successfully reduced common method bias. Self-report measures are also prone to social desirability bias, where participants respond in a manner they perceive as socially acceptable or pleasing to the researchers. Study instructions countered this by stating that there were no correct or desired responses and answers should reflect the opinions of individual participants. To extend the current paper subsequent investigations could triangulate self-report measure scores with objective measures. This is particularly important in the context of reasoning measures because self-report relies on accurate metacognition, and individuals often have biased or inaccurate perceptions of their own cognitive processes and abilities. This is especially for reasoning processes as they are not fully consciously accessible. Hence individuals may lack full awareness of their own cognitive processes and biases. This can lead to discrepancies between reported (subjective) and actual reasoning performance (objective).

Due to the complex nature of critical thinking, which encompasses a range of skills, assessing a participant’s analytical thinking preferences requires a multi-method approach. For example, the Watson-Glaser Critical Thinking Appraisal (WGCTA; Watson and Glaser, 1980) assesses multiple components, including recognition of assumptions (identifying unstated beliefs), evaluation of arguments (assessing the strength and relevance of statements), inference (drawing correct conclusions), interpretation (considering evidence for generalizations), and deduction (determining whether conclusions follow logically from premises).

Additionally, individual assessments measure only certain aspects of complex reasoning processes. For this reason, comparisons both across and within tools that evaluate analytical-rational and intuitive-experiential engagement are necessary to build a complete and nuanced picture of cognitive style and critical thinking proficiency.

Secondly, the use of social media to recruit participants potentially limited the generalizability of findings. While this is a commonly used method within psychology, it samples only individuals who engage with social media platforms. Hence, samples might not be representative of the broader population in terms of demographics, interests, or online engagement habits.

Moreover, participants were self-selecting sample. This introduces potential for sampling bias. For instance, individuals with a high degree of distrust, hostility, or indifference toward science may have been less inclined to volunteer for a survey focusing on scepticism and belief in science, resulting in an underrepresentation of extreme lower evidence-based thinkers. Conversely, the research topic may have disproportionately attracted individuals with a strong intellectual interest and motivation to express their views (overly engaged). Such factors could skew belief profile distribution compared to the general population.

Additionally, with approximately 87.6% of participants identifying as White British/Irish or White other, the sample exhibited a lack of ethnic diversity. This overrepresentation of an ethnic group means that the identified profiles may not prove representative of the broader population. Hence, finding generalization to diverse ethnic and cultural contexts requires caution. For these reasons ensuing investigations should replicate the outcomes of this study using more representative sample groups. Larger more heterogeneous samples will also enable demographic comparisons to determine the extent to which auxiliary characteristics influence scepticism. Furthermore, it remains to be seen if these distinct profiles persist in non-Western or culturally diverse contexts, where the boundaries between scientific and supernatural epistemic stances may be more fluid than in the current sample.

Thirdly, the study employed a cross-sectional design, meaning the researchers collected data at a single point in time. This prevented establishment of causal relationships between belief systems and cognitive processing styles. To address this concern, subsequent investigations should employ multiple measurement and/or longitudinal designs, which will enable exploration of developmental pathways across assessments points and time enabling the establishment of causal inferences.

Fourthly, a limitation of LPA is that profile identification is a function of variable heterogeneity within the sample assessed. Consequently, profile number and nature may vary across populations. To ensure consistency and generalizability, future investigations must employ replication and cross-validation methods such as progressive elaboration (Donovan and Chung, 2015). This approach will protect interpretation from misspecification, establish class stability and model fit, and facilitate the development of robust, theoretically sound subgroups (Collins et al., 1994). Nonetheless, the profiles revealed in this study remain valuable since they advance conceptual understanding of scepticism (Drinkwater et al., 2024a, 2024b).

Finally, although the study employed established measures of paranormal and scientific beliefs, carefully operationalizing these constructs for precision, it is important to note that the instruments assessed only specific elements of complex, multi-faceted phenomena. In the case of the Belief in Science Scale, the instrument focuses on determining the extent to which respondents perceive science as an exclusively valuable and superior guide to reality (Farias et al., 2013). The primary limitation of the Belief in Science Scale is the instrument’s focus on a single, overarching dimension. This neglects other important aspects of scientific understanding such as appreciation of processes/conventions, trust in institutions, and acceptance of scientific consensus.

With reference to the Revised Paranormal Belief Scale, though the measure captures a breadth of supernatural phenomena, it omits important aspects of paranormality, such as ghosts/hauntings, poltergeists, folklore entities, or other culturally relevant phenomena not covered by its established subscales. Hence, additional work needs to examine the degree to which the findings of the present study extend to these supplementary facets of paranormal and scientific belief. Additionally, to ensure generalizability, future research should consider cultural variations in the appreciation and understanding of scientific and paranormal beliefs.

## Conclusion

This study provides empirical support for the notion that scientific scepticism and paranormal beliefs are associated with distinct, oppositional cognitive processing styles. By identifying two latent profiles, Higher Evidence-based Thinking and Lower Evidence-based Thinking, the findings highlight how individual differences in preferential processing influence the endorsement of paranormal and scientific claims.

Significantly, the results move beyond the traditional “deficit model” by demonstrating that cognitive rigidity and epistemic closure are general features of strongly held beliefs rather than specific to any one epistemic orientation. This research underscores the importance of viewing scientific and paranormal worldviews as competing cognitive structures, each driven by unique processing preferences that determine the validation of incoming information.

## Data Availability

The raw data supporting the conclusions of this article will be made available by the authors, without undue reservation.
