# Supplementary material for: Mind over matter? The cognitive styles of scientific scepticism and paranormal belief
Source: Front Psychol. 2026 Mar 6;17:1699045. doi: 10.3389/fpsyg.2026.1699045 (PMC13002858; doi:10.3389/fpsyg.2026.1699045)
Supplement: Supplementary file 1 [file Table_1.DOCX]

**Supplementary Table 1** Demographic characteristics of the sample

|  | Category | Number | % of the sample |
| --- | --- | --- | --- |
| Gender | Male | 100 | 33.3 |
|  | Female | 187 | 62.3 |
|  | Non-binary | 8 | 2.7 |
|  | Prefer not to say | 5 | 1.7 |
| Ethnicity | African | 3 | 1.0 |
|  | Black other | 1 | .3 |
|  | Person of colour British / Irish | 1 | .3 |
|  | Person of colour other | 4 | 1.3 |
|  | South Asian | 1 | .3 |
|  | Southeast Asian | 2 | .7 |
|  | White British / Irish | 208 | 69.3 |
|  | White other | 55 | 18.3 |
|  | Other | 24 | 8.0 |
|  | Prefer not to say | 1 | .3 |
| Nationality | African | 1 | .3 |
|  | Asian | 3 | 1.0 |
|  | Australasian | 3 | 1.0 |
|  | European | 21 | 7.0 |
|  | North American | 61 | 20.3 |
|  | Ireland | 2 | .7 |
|  | United Kingdom | 192 | 64.0 |
|  | Other | 15 | 5.0 |
|  | Prefer not to say | 2 | .7 |
| Education | PhD and post-doctorate | 26 | 8.7 |
|  | Masters / postgraduate | 75 | 25.0 |
|  | Bachelor’s degree | 75 | 25.0 |
|  | Accredited professional training qualification | 23 | 7.7 |
|  | Nationally recognised diploma or equivalent | 28 | 9.3 |
|  | Completion of apprenticeship (or equivalent) | 1 | .3 |
|  | ‘A’ Levels (or equivalent if outside of the UK) | 33 | 11.0 |
|  | ‘O’ Levels / GCSEs (or equivalent if outside of the UK) | 18 | 6.0 |
|  | Other | 11 | 3.7 |
|  | No qualifications | 4 | 1.3 |
|  | Prefer not to say | 6 | 2.0 |
| Income | Comfortable with extra | 117 | 39.0 |
|  | Enough but no extra | 124 | 41.3 |
|  | Have to cut back | 41 | 13.7 |
|  | Cannot make ends meet | 12 | 4.0 |
|  | Prefer not to say | 6 | 2.0 |
